# Digital photography provides a fast, reliable, and noninvasive method to estimate anthocyanin pigment concentration in reproductive and vegetative plant tissues

**DOI:** 10.1002/ece3.3804

**Published:** 2018-02-16

**Authors:** José C. del Valle, Antonio Gallardo‐López, Mª Luisa Buide, Justen B. Whittall, Eduardo Narbona

**Affiliations:** ^1^ Department of Molecular Biology and Biochemical Engineering Pablo de Olavide University Seville Spain; ^2^ Department of Biology Santa Clara University Santa Clara CA USA

**Keywords:** anthocyanins, color measurement, image calibration, image processing, intraspecific variation, pigment quantification, pigmentation pattern, spectral reflectance

## Abstract

Anthocyanin pigments have become a model trait for evolutionary ecology as they often provide adaptive benefits for plants. Anthocyanins have been traditionally quantified biochemically or more recently using spectral reflectance. However, both methods require destructive sampling and can be labor intensive and challenging with small samples. Recent advances in digital photography and image processing make it the method of choice for measuring color in the wild. Here, we use digital images as a quick, noninvasive method to estimate relative anthocyanin concentrations in species exhibiting color variation. Using a consumer‐level digital camera and a free image processing toolbox, we extracted RGB values from digital images to generate color indices. We tested petals, stems, pedicels, and calyces of six species, which contain different types of anthocyanin pigments and exhibit different pigmentation patterns. Color indices were assessed by their correlation to biochemically determined anthocyanin concentrations. For comparison, we also calculated color indices from spectral reflectance and tested the correlation with anthocyanin concentration. Indices perform differently depending on the nature of the color variation. For both digital images and spectral reflectance, the most accurate estimates of anthocyanin concentration emerge from anthocyanin content‐chroma ratio, anthocyanin content‐chroma basic, and strength of green indices. Color indices derived from both digital images and spectral reflectance strongly correlate with biochemically determined anthocyanin concentration; however, the estimates from digital images performed better than spectral reflectance in terms of *r* ^2^ and normalized root‐mean‐square error. This was particularly noticeable in a species with striped petals, but in the case of striped calyces, both methods showed a comparable relationship with anthocyanin concentration. Using digital images brings new opportunities to accurately quantify the anthocyanin concentrations in both floral and vegetative tissues. This method is efficient, completely noninvasive, applicable to both uniform and patterned color, and works with samples of any size.

## 1 INTRODUCTION

Apart from chlorophylls, anthocyanins are one of the main pigments conferring color in plants, being almost ubiquitous among angiosperms (Tanaka, Sasaki, & Ohmiya, 2008). Anthocyanins may be accumulated in all organs and are usually stored in vacuoles of the epidermis or mesophyll (Gould, Davies, & Winefield, 2008; Lee, O'Keefe, Holbrook, & Feild, 2003; Wheldale, 1916). In flowers and fruits, anthocyanins confer colors ranging from orange to red to blue to purple, whereas in vegetative organs, mostly red or purple colors are observed (Lee, 2007). The pigments absorb light at specific wavelengths, and the remaining light is reflected or scattered by plant structures, such as vacuoles or epidermal cells, which produces the visible colors in wavelengths spanning 400 to 700 nm (van der Kooi, Elzenga, Staal, & Stavenga, 2016; Lee, 2007). The manner in which anthocyanins affect final pigmentation mainly depends on the type of anthocyanin(s) that accumulates and its concentration, but their color can also be influenced by the type and amount of linked co‐pigment, metals, and pH (Gonnet, 1999; Tanaka et al., 2008).

Anthocyanins provide adaptive benefits for many plants (reviewed in Archetti et al., 2009; Landi, Tattini, & Gould, 2015; Strauss & Whittall, 2006). In reproductive organs, anthocyanins help attract pollinators or seed dispersers, whereas in vegetative organs, they provide protection against environmental stressors such as UV‐B radiation, excess light, cold, drought, salinity, pathogens, and/or herbivores (Landi et al., 2015; Lee & Gould, 2002; Schaefer & Ruxton, 2011). Variation in floral and vegetative anthocyanin concentrations within and among populations is common and often adaptive (e.g., Del Valle, Buide, Casimiro‐Soriguer, Whittall, & Narbona, 2015; Menzies et al., 2016). Therefore, it is undeniable that the quantification of anthocyanins has become fundamental in understanding many aspects of plant evolutionary ecology (Ortiz‐Barrientos, 2013; Sobel & Streisfeld, 2013).

A battery of methods has been used to quantify the amount of anthocyanin pigment present in plant tissues. Pigment extraction by wet chemical methods in organic solvents and subsequent HPLC or spectrophotometric quantification is the most frequently used (Abdel‐Aal & Hucl, 1999). Although these biochemical methods are extremely accurate (Lee, Rennaker, & Wrolstad, 2008), they are also time‐consuming and expensive; more important, they consume the tissues measured, limiting investigation of other aspects of color (e.g., pollinator preference, fitness, etc.). Some remarkable alternatives to biochemical methods are based on UV–Vis spectral reflectance (Gamon & Surfus, 1999; Gitelson, Chivkunova, & Merzlyak, 2009). Analysis of reflected wavelength distribution through digital portable spectrophotometers following the use of appropriate indices for the specific pigment has become a widely used methodology to estimate relative pigment concentration in leaves and fruits (Gitelson et al., 2009; Merzlyak, Solo, & Gitelson, 2003; Richardson, Duigan, & Berlyn, 2002; Sims & Gamon, 2002) and even in petals (Narbona & Whittall, unpublished data). Because of the increasing portability of spectrophotometers and the small size of the measurable plant area, this method can be considered noninvasive (Gamon & Surfus, 1999; Richardson et al., 2002). However, taking spectral reflectance of delicate plant parts such as petals or small leaves, the tissue usually has to be removed from the plant or at the least is usually damaged (see also Bergman & Beehner, 2008).

On the other hand, digital photography is a fast, noninvasive alternative which has become the method of choice for measuring color both in animals and in plants (Bergman & Beehner, 2008; Garcia, Greentree, Shrestha, Dorin, & Dyer, 2014; Kendal et al., 2013; Mizunuma et al., 2014; Stevens, Lown, & Wood, 2014). With relatively simple camera settings, a few precautions before taking the photograph, and easy image processing (Stevens, Párraga, Cuthill, Partridge, & Troscianko, 2007; Troscianko & Stevens, 2015; White et al., 2015), digital imaging is an efficient and reliable method to quantify color, even in the field (Bergman & Beehner, 2008; Macfarlane & Ogden, 2012; Stevens et al., 2014). Recently, there have been several intriguing applications of digital photography to study plant and animal coloration, such as assessment of color change, pigment patterns, and camouflage (Akkaynak et al., 2014; Gómez & Liñán‐Cembrano, 2016; Stevens et al., 2014; Strauss & Cacho, 2013; Taylor, Gilbert, & Reader, 2013). In spite of these advantages, the use of digital images to faithfully quantify color variation in plants is in its infancy. As far as we know, the application of digital images to estimating pigment concentration has only been studied in leaves of sugar maple, which undergo dramatic, seasonal changes in pigment composition (Junker & Ensminger, 2016).

In this study, we describe an efficient, noninvasive method for estimating relative anthocyanin concentration using digital images. Our method is based on current knowledge of image processing to measure plant color (Troscianko & Stevens, 2015), incorporating the application of new indices related to output data from digital images of plants. Our objective is to determine the suitability of our method for different plant tissues and pigmentation patterns. Thus, we assessed anthocyanins in petals that accumulate anthocyanin pigments, as well as in other plant parts such as pedicels, calyces, and stems which also contain chlorophylls, from a total of six species. Each plant tissues studied showed variation in color intensity and pigmentation pattern (uniform, striped, spotted, and with veins). The digital image method was assessed by comparison with the color indices with anthocyanin concentration quantified biochemically. Color indices based on spectral reflectance were also compared to anthocyanin concentrations. Most anthocyanins are characterized by an absorption maxima in the ~520–560 nm region (Merken & Beecher, 2000). Although UV reflectance may occur in the flowers of some plant species (Glover, 2007; Koski & Ashman, 2016), this is primarily caused by other flavonoids such as flavones, flavonols, and flavanones (Merken & Beecher, 2000). These nonanthocyanin flavonoids do not absorb in the visible region; thus, they not interfere in the anthocyanin estimates although can acts as co‐pigments. Herein, to estimate anthocyanin concentration, we focus on the use of digital images to capture data from the visible region of the spectrum.

## 2 MATERIALS AND METHODS

### 2.1 Plant materials and sampling

**Figure 1:**
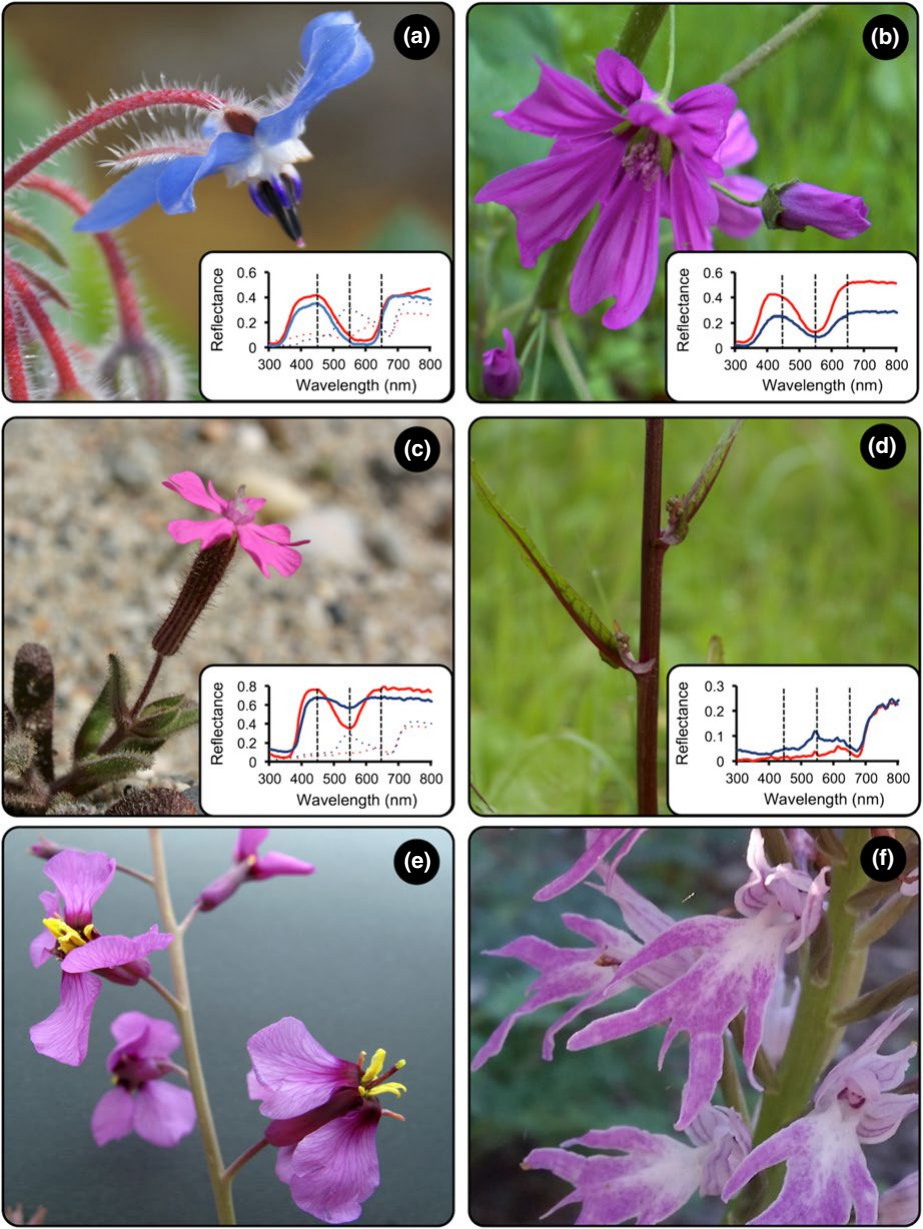
Photographs of the species and tissues considered in the estimation of anthocyanin concentration with digital images showing the diversity of colors and pigmentation patterns. Spectral reflectances are included for the species that anthocyanin concentration was also estimated by portable spectrophotometer. Red and blue solid lines are the darkest and lightest samples of petals, respectively. Red and blue dotted lines represent the darkest and lightest samples of the other studied tissues. (a) Petals and pedicels of *Borago officinalis*. (b) Petals of *Malva sylvestris*. (c) Petals and calyces of *Silene littorea*. (d) Stems of *Sonchus oleraceus*. (e) Petals of *Moricandia moricandioides*. (f) Petals of *Orchis italica*

In spring of 2016, we collected one flower from 19 to 41 individuals of one or two populations for each of five species in southern Spain belonging to phylogenetically diverse angiosperm groups: *Borago officinalis* L., *Malva sylvestris* L., *Moricandia moricandioides* (Boiss.) Heywood, *Orchis italica* Poir. and *Silene littorea* Brot. (Table 1). We sampled flowers in early anthesis, representing the maximum range of color variation. We focused on flower parts containing anthocyanin pigments; thus, we analyzed petals (or labellum in *O. italica*) for all species except for *B. officinalis* and *S. littorea* for which we also collect pedicels (approx. 1 cm long) and calyces, respectively (Figure 1). In addition, we studied stems of *Sonchus oleraceus* L., from which 1 cm of the main stem was cut with a razor blade. For four of the six sampled species (*B. officinalis*,* M. sylvestris*,* S. littorea*, and *S. oleraceus*), we estimated the anthocyanin concentration by three methods: digital images, spectral reflectance, and biochemistry. Immediately after collecting the plant material, we captured a digital image, measured the spectral reflectance, and placed the plant material into a 1.5 ml of MeOH:HCl (99:1% v:v). Because petals are damaged in the process of measuring spectral reflectance, we used one petal from each flower for this method, leaving the remaining petals of each flower for the other two methods. For the remaining species (*M. moricandioides* and *O. italica*), only digital images and biochemical methods were assessed.

**Table 1:** Species and plant tissues analyzed in this study

Species (Family)	Plant part (*N*)	Color (pattern)	Anthocyanin type	λ_max_	Anthocyanin concentrations (AU. cm^−2^)		Size of sample area (mm^2^)	Pixels per unit area (pixels/mm^2^)
					Range	CV (%)		
*Borago officinalis* (Boraginaceae)	Petals (23)	Blue (uniform)	Del‐3,5‐G; Pet‐3,5‐G [a]	530	0.09–0.23	26.4	17.6–18.1	2624.1–2873.8
	Pedicels (19)	Red (uniform, hairy)	Unknown	535	0.00–1.03	126.0	7.9–14.9	1474.9–1664.8
*Malva sylvestris* (Malvaceae)	Petals (26)	Mauve (striped)	Mal‐3,5‐G [b]	535	0.03–0.25	48.8	103.6–134.8	2623.5–2714.2
*Moricandia moricandioides* (Brassicaceae)	Petals (41)	Purple (venation)	Peo‐3‐S,5‐G; Cya‐3‐S,5‐G [c]	560	0.05–0.20	30.2	28.7–97.7	1464.3–1536.7
*Orchis italica* (Orchidaceae)	Petals (19)	Pink (spotted)	Cya‐3‐G [d]	530	0.06–0.50	60.8	24.6–57.8	1602.6–4735.6
*Silene littorea* (Caryophyllaceae)	Petals (28)^a^	Pink (uniform)	Cya‐3‐G [e]	520	0.04–0.33	51.5	2.8–64.3	2869.4–3796.9
	Calyces (29)	Red (striped)	Cya‐3‐G [f]	520	0.04–0.72	67.2	19.8–27.5	2539.7–4410.3
*Sonchus oleraceus* (Asteraceae)	Stems (31)	Red (uniform)	Cya‐3‐G [g]	530	0.01–0.98	74.5	26.2–110.1	1867.0–2227.7

For each plant tissue, coloration pattern, main type of anthocyanin pigment accumulated, wavelength used to biochemically quantify anthocyanins (λ_max_), descriptive statistics of relative anthocyanin concentration, minimum and maximum size values of sample area and number of pixels per unit area measured from digital images are shown. References for anthocyanin pigment identification are showed in square brackets.

AU, absorbance units; CV, coefficient of variation; Del, delphinidin; Pet, petunidin; Mal, malvidin; Peo, peonidin; Cya, cyanidin; G, glucoside; S, sophoroside.

a Another 28 independent samples were used to model validation (see methods section). [a] Salem et al. (2014); [b] Farina et al. (1995); [c] Tatsuzawa et al. (2012); [d] Strack, Busch, and Klein (1989); [e] Casimiro‐Soriguer, Narbona, Buide, del Valle, and Whittall (2016); [f] Alcalde‐Eon and Del Valle unpublished data; [g] Price and Sturgess (1938).

### 2.2 Anthocyanin quantification by biochemistry

After samples were placed in microcentrifuge tubes, they were temporarily stored for 1–2 hr in the dark at room temperature and then frozen at −80°C until subsequent anthocyanin extraction and biochemical quantification (1–3 months later). Due to the thinness of the petal samples, the MeOH:HCl solution was sufficient to completely extract all anthocyanins. Thus, we only removed the transparent petals from the methanol extract before anthocyanin quantification. For complete anthocyanin extraction in pedicels, calyces, and stems samples, we used the methods described in Del Valle et al. (2015).

Three replicates of 200 μl per sample were measured in a Multiskan GO microplate spectrophotometer (Thermo Fisher Scientific Inc., MA, USA). Previously, extracts of each species and plant tissues were scanned across visible wavelengths to identify the maximum absorbance (*A*_max_) of anthocyanins; this wavelength was confirmed with the literature whenever possible (Table 1). Some species with similar anthocyanin derivatives showed different maximum wavelength absorption due to possible effects of co‐pigments, and the number, position, and identity of glucosides linked to the anthocyanidin skeleton (Andersen & Jordheim, 2006; Brouillard & Dangles, 1994). In photosynthetic tissues, anthocyanin concentration was corrected using the equation *A*_max_ − 0.24*A*_653_ (Murray & Hackett, 1991). Total amounts of anthocyanins were expressed as absorbance units (AU) per cm^2^ of fresh material.

### 2.3 Color spectra measurements

UV–vis spectral reflectance of each sample was measured with a Jaz portable spectrometer (Ocean Optics Inc., Dunedin, FL, USA) equipped with a Deuterium–Tungsten halogen light source (200–2,000 nm) and a black metal probe holder (6 mm diameter opening at 45°). Reflectance, relative to a white standard WS‐1‐SL, was analyzed with SpectraSuite v.10.7.1 software (Ocean Optics). In order to maximize the amount of light used in reflectance measurements and reduce occasionally erratic reflectance values at individual nm, we set an integration time of 2 s and smoothing boxcar width of 12, respectively (White et al., 2015). To calculate different color indices, we analyzed spectral wavelengths from 300 to 800 nm at 0.4 nm intervals.

### 2.4 Digital images

Prior to using digital images to estimate anthocyanin content, one must confirm experimentally or using the literature (e.g., Andersen & Jordheim, 2006; Harborne, 1994) that the pigment(s) underlying the color of their samples are anthocyanins. The method presented here was tested for samples containing exclusively anthocyanins or anthocyanins with nonanthocyanin flavonoids or chlorophylls; the additional accumulation of carotenoids could significantly affect the tissue color and thus introduce errors in any subsequent estimation of anthocyanin concentration.

**Figure 2:**
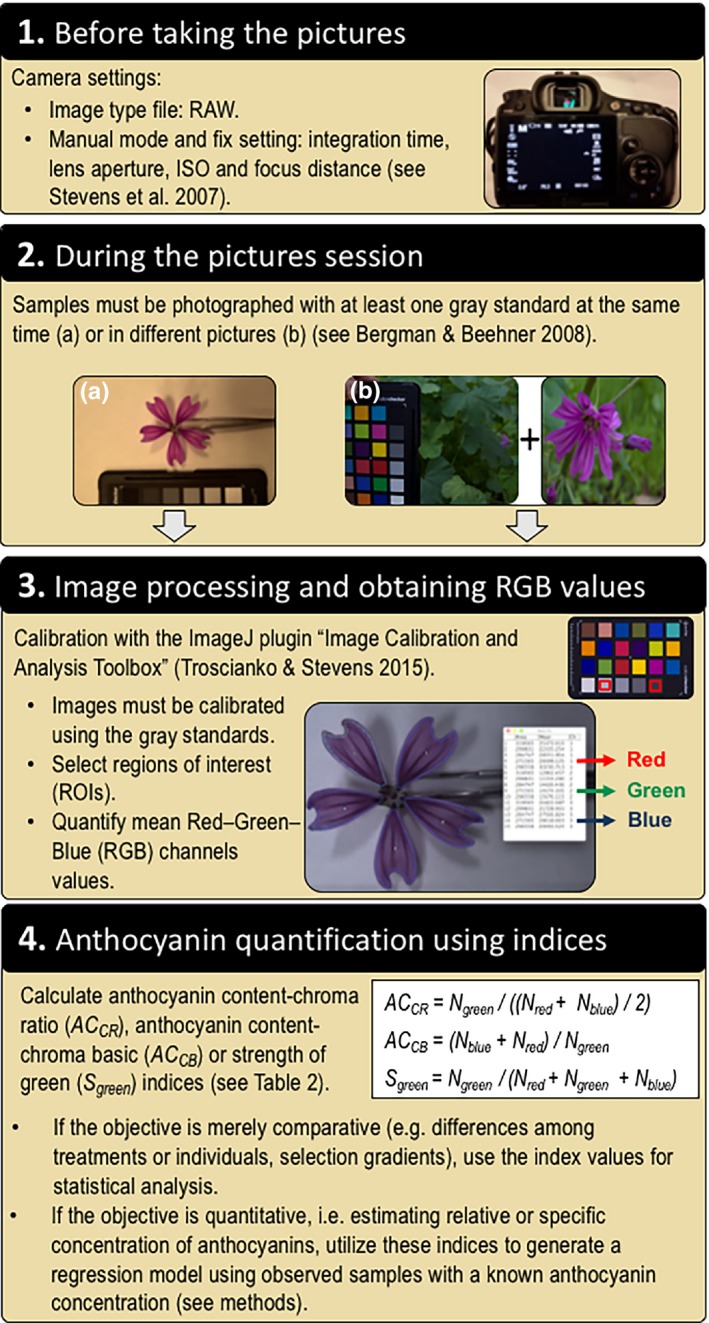
Diagrammatic representation of the steps required to estimate anthocyanin concentration from digital images. Note that this method is tested for samples containing exclusively anthocyanins or anthocyanins with chlorophylls or nonanthocyanin flavonoids

The four steps necessary to quantify the anthocyanin concentration from digital images are depicted in Figure 2. First, we used a Sony α65 DSLR camera (Sony Corporation, Tokyo, Japan) equipped with a Sam 18–55 mm autofocus lens (transmitting wavelengths of 400–700 nm). This camera has a 23.5 × 15.6 mm CMOS sensor (6,024 × 4,024 pixels) and shows full regulation of exposure and metering, as recommended for unbiased data acquisition (Stevens et al., 2007; White et al., 2015). We manually adjusted these settings for all samples: lens aperture of f/5.6, ISO 100, and white balance fixed at 4500k; the exception was integration time, set for each species from 1/30 to 1/100, depending on specific light conditions. We deliberately underexposed all photographs by 0.3 f‐stop to prevent color “clipping” or saturation (Stevens et al., 2007). Images were taken in Sony Alpha RAW format (ARW). RAW files are the recommended format because they contain unprocessed images which may be linearized using specialized software (see Figure 2, step 3).

Second, each sample was photographed with a ColorChecker Passport (X‐Rite Inc., Grand Rapids, MI) for standardization across light conditions (Figure 2, step 2). With calyces of *S. littorea* we used the “sequential method”: We photographed the ColorChecker chart and then performed a series of 5–8 photographs of plant samples under the same light conditions as the chart (Figure 2, step 2b; Bergman & Beehner, 2008; Troscianko & Stevens, 2015). Photographs were taken under natural light condition, but in the shade, to prevent shadow and excessive brightness (Kendal et al., 2013).

Third, we calibrated digital photographs to allow their use for objective measurements of color or pattern within or between photographs (Akkaynak et al., 2014; Stevens et al., 2007). For image processing, we used the freeware “Image Calibration and Analysis Toolbox” (Troscianko & Stevens, 2015), which is a plugin for ImageJ software (Schneider, Rasband, & Eliceiri, 2012). The major advantages of this toolbox are easy linearization, high precision, and low data loss in image analysis due to 32‐bit floating‐point image processing (Troscianko & Stevens, 2015). For image calibration, we selected two gray standards of the ColorCheckert (Neutral 3.5 and Neutral 8, with 9.11% and 60.90% reflectance, respectively; Myers, 2010) and used the setting for “visible” photography and “aligned normalized 32‐bit” files. Calibrations performed using these two gray standards produce statistically similar results when compared to calibrations with all six gray standards (Table S1). The calibration process successfully linearized the RGB values (Figure S1). Regions of interest (ROIs) were selected in each image (i.e., specific areas of plant tissue analyzed), and the mean values of red–green–blue (RGB) channels were extracted. Following the recommendations of White et al. (2015), the size of sampled area and the number of pixels per unit area are shown in Table 1.

### 2.5 Color indices and statistical analyses

Four, we used the RGB values in several indices to analyze colors and estimate pigment concentration. In the literature, there are a myriad of indices that can be obtained from color spectra data (Endler, 1990; Gitelson et al., 2009; Gomez, 2006; Montgomerie, 2006) and digital image data (i.e. RGB values; Gillespie, Kahle, & Walker, 1987; Woebbecke, Meyer, Von Bargen, & Mortensen, 1995; Mizunuma et al., 2014). For spectral reflectance, we used indices related to the physical properties of light which are independent of the observer's visual system, except for segment analysis indices, chosen because it compares different regions of the wavelength spectra (Endler, 1990; Kemp et al., 2015). In addition, we proposed two new indices which consider the shape of the spectra with two peaks at 450 and 650 nm and a minimum at approximately 550 nm (Figure 1), and other five new indices for digital images comparing the G against the R and B channels; all indices including those newly developed are described in Table 2.

**Table 2:** Color indices used to estimate anthocyanin concentration from spectral reflectance and digital image data

Color indices	Formula used for spectral reflectance	Formula used for digital images
Hue	*H* = λ *R*_max_ [a]	*H* = (*g* − *b*)/((*I*_max_ − *I*_min_) × 60) [h] *H′* = (2 * *r *–* g *−* b*)/(*g *−* b*) [i]
Hue‐segment classification	*H*_SC_ = sign ($\sum_{500}^{599}R_{i}-\sum_{300}^{399}R_{i}$) * arcsine (($\sum_{600}^{699}R_{i}-\sum_{400}^{499}R_{i}$)/*C*_SC_) modulus 2 π [b]	
Brightness	$B=\sum_{300}^{699}R_{i}$ [a]	$B=\surd \left[\left(b^{2}+g^{2}+r^{2}\right)/3\right]$ [i]
Lightness		*L* = (*I*_max_ + *I*_min_)/2 [h]
Saturation		*S* = (*I*_max_ − *I*_min_)/(2 − (*I*_max_ + *I*_min_)) [h]
Chroma	*C* = (*R*_max_ − *R*_min_)/*R*_average_ [c]	*C *= (*N*_red_ − *N*_green_)/((*N*_red_ + *N*_green_ + *N*_blue_)/3) [e] $C^{\prime }-\surd \left({\left(N_{\mathrm{red}}-N_{\mathrm{green}}\right)}^{2}+{\left(N_{\mathrm{blue}}-N_{\mathrm{green}}\right)}^{2}\right)$ [e]
Chroma‐segment classification	$\begin{array}{c} C_{\mathrm{SC}}=\surd \left({\left(\sum_{600}^{699}R_{i}-\sum_{400}^{499}R_{i}\right)}^{2}+{\left(\sum_{500}^{599}R_{i}-\sum_{300}^{399}R_{i}\right)}^{2}\right) \end{array}$ [a]	
Anthocyanin content‐chroma difference	*AC*_CD_ = ((*R*_450_ + *R*_650_)/2) − *R*_550_ [d]	*AC*_CD_ = ((*N*_blue_ + *N*_red_)/2) − *N*_green_ [e]
Anthocyanin content‐chroma ratio	*AC*_CR_ = *R*_550_/((*R*_450_ + *R*_650_/2) [e]	*AC*_CR_ = *N*_green_/((*N*_blue_ + *N*_red_)/2) [e]
Anthocyanin content‐chroma basic	*AC*_CB_ = (*R*_450_ + *R*_650_)/*R*_550_ [e]	*AC*_CB_ = (*N*_blue_ * *+* N*_red_)/*N*_green_ [e]
Red:green ratio	$R:G_{R}=\sum_{600}^{699}R_{i}/\sum_{500}^{599}R_{i}$ [f]	*R:G*_R_ = *N*_red_/*N*_green_ [j]
Red:green index	$R:G_{I}=\sum_{690}^{710}R_{i}/\sum_{540}^{560}R_{i}$ [g]	
Modified anthocyanin content index	$\text{mACI}=\sum_{760}^{800}R_{i}/\sum_{540}^{560}R_{i}$ [g]	
Strength of green	$S_{\mathrm{green}}=\sum_{545}^{565}R_{i}/\left(\sum_{620}^{670}R_{i}+\sum_{545}^{565}R_{i}+\sum_{459}^{479}R_{i}\right)$ [h]	*S*_green_ = *N*_green_/(*N*_red_ + *N*_green_ + *N*_blue_) [h]
Strength of red	$S_{\mathrm{red}}=\sum_{620}^{670}R_{i}/\left(\sum_{620}^{670}R_{i}+\sum_{545}^{565}R_{i}+\sum_{459}^{479}R_{i}\right)$ [h]	*S*_red_ = *N*_red_/(*N*_red_ + *N*_green_ + *N*_blue_) [h]
Strength of blue	$S_{\mathrm{blue}}=\sum_{459}^{479}R_{i}/\left(\sum_{620}^{670}R_{i}+\sum_{545}^{565}R_{i}+\sum_{459}^{479}R_{i}\right)$ [h]	*S*_blue_ = *N*_blue_/(*N*_red_ + *N*_green_ + *N*_blue_) [h]

References shown in square brackets.

λ = Wavelength (nm); *R*_*i*_ = Reflectance, relative to white standard, in wavelength *i*;* N*_red_, *N*_green_ and *N*_blue_ are values of red, green, blue channels, respectively; *r, b, g * =  values of each channel divided by the total number of possible values for the channel (i.e. 65,535 for 16‐bit images); *I*_max_ and *I*_min_ are maximum and minimum values of *r*,* g* and *b*. [a] Endler (1990); [b] Smith (2014); [c] Andersson, Pryke, Örnborg, Lawes, and Andersson (2002); [d] Frey (2004); [e] New indices proposed in this study; [f] Gamon and Surfus (1999); [g] Gitelson et al. (2009); [h] Mizunuma et al. (2014); [i] Mathieu, Pouget, Cervelle, and Escadafal (1998); [j] Bergman and Beehner (2008).

Because our primary goal is to test whether anthocyanin concentration can be predicted from indices obtained from digital images versus spectral reflectance and the strength of this relationship, we used least‐squares linear regressions (Warton, Wright, Falster, & Westoby, 2006). Preliminary graphic inspection showed that our data were appropriate for simple linear regressions. In some cases, variables were log transformed to meet requirements for normality of residuals and homoscedasticity (Crawley, 2012). To measure goodness of fit of the regression models, the explained variance (*r* ^2^) was used. We applied the sequential Bonferroni test to control for experiment‐wide type I error produced by the fourteen indices we compared (*P *< α/14 for each dataset; Rice, 1989).

To compare the accuracy of the model's predictions between the spectral reflectance and the digital image methods, we used the normalized root‐mean‐square error (NRMSE). Normalization was performed by dividing RMSE by the maximum variability (maximum minus minimum value) in the observed data, which allows comparisons among models with different scales of variables (Willmott, 1981). NRMSE were calculated in the four common indices that best fit our data (anthocyanin content‐chroma basic (*AC*_CB_), anthocyanin content‐chroma ratio (*AC*_CR_), *R:G*_R_ and strength of green (*S*_green_); see Section 2.1). To additionally assess how our regression model predicts the amounts of anthocyanins, we performed a model validation using independent data from *S. littorea* petals (Data S1).

All analyses were performed with R version v3.1.1 (R Core Team 2016), and graphs were created with R‐package ggplot2 v2.0.0 (Wickham, 2009).

## 3 RESULTS

For petals, interindividual variation for the relative amount of anthocyanin measured in terms of the percent coefficient of variation ranged from 26% in *B. officinalis* to 61% in *O. italica* (Table 1). Photosynthetic tissues showed higher levels of variation among samples, with coefficients of variation ranging from 67% in the calyces of *S. littorea* to 126% in the pedicels of *B. officinalis*.

Results of linear relationships between relative anthocyanin concentration and indices calculated from digital images are shown in Table 3. *H* presented weak or nonsignificant relationships in petals of all species except *O. italica*, whereas in the rest of tissues, the association was moderate‐high (*r* ^2^ = .60–.93). *H*′ showed similar results to *H*, but in the case of *O. italica* petals and *S. oleraceus* stems, the relationship was nonsignificant. *B*,* L*, and *S* indices displayed different results, in some samples performed well, whereas in others had nonsignificant or weak relationships. In general, chroma and anthocyanin content indices showed similar coefficients of determination and performed very well for all species and tissues (higher *r* ^2^ = .80–.93) and moderate‐well for *S. littorea* calyces (higher *r* ^2^ = .65; Table 3). A special case was found in *B. officinalis* petals, with moderate or nonsignificant relationships in *R:G*_R_, *AC*_CD_, and *C*′ indices. This is because their blue petals had higher values in the blue and green channels than the red one, generating values between 0 and 1, which reduces absolute differences between samples. Among indices related to strength of RGB channels, *S*_green_ had the best relationship with relative anthocyanin concentration, which had similar predictive power to those found in anthocyanin content indices. The indices showing the highest *r* ^2^ were as follows: *S*_green_ (five studied samples), *AC*_CB_ (four studied samples), *AC*_CR_ (three studied samples), and *R:G*_R_ (two studied samples; Table 3).

**Table 3:** Coefficient of determination (*r* ^2^) and statistical significance of linear regressions between relative anthocyanin concentration and digital image indices

Indices	*B. officinalis*	*B. officinalis*	*M. sylvestris*	*M. moricandioides* a	*O. italica* a	*S. littorea*	*S. littorea*	*S. oleraceus* a
	Petals	Pedicels	Petals	Petals	Petals	Petals	Calyces	Stems
Hue (*H*)	0.01ns	0.93***	0.06ns	0.36***	0.68***	0.33*	0.61***	0.60***
Hue (*H′*)	<0.01ns	0.92***^,^ a	<0.01ns	0.36***	0.05ns	0.28ns	0.55***^,^ a	0.03ns
Brightness (*B*)	0.20ns	0.77***	0.75***	0.75***	0.16ns	0.43**	0.19ns	0.45***^,^ a
Lightness (*L*)	0.26ns	0.71***	0.77***	0.79***	0.17ns	0.42**	0.14ns	0.36**^,^ a
Saturation (*S*)	0.66***	0.28ns	0.85***	0.83***	0.10ns	0.87***	0.31*	0.28*
Chroma (*C*)	0.38*	0.79***^,^ a	0.85***	0.82***	0.73***	0.87***	0.56***	0.78***
Chroma (*C′*)	0.07ns	0.57**	0.70***	0.25*	0.51**	0.65***	0.40**	<0.01ns
Anthocyanin content‐chroma difference (*AC*_CD_)	0.11ns	0.89***	0.69***	0.28**	0.84***	0.67***	0.50***	0.59***
Anthocyanin content‐chroma ratio (*AC*_CR_)	0.82***	0.93***	0.88***^,^ a	0.78***	0.86***	0.88***	0.62***	0.80***^,^ a
Anthocyanin content‐chroma basic (*AC*_CB_)	0.83***	0.92***	0.88***	0.75***	0.85***	0.88***	0.65***	0.78***
Red:green ratio (*R:G*_R_)	0.46**	0.90***	0.87***	0.83***	0.75***	0.88***	0.58***	0.75***
Strength of green (*S*_green_)	0.82***	0.94***	0.88***	0.77***	0.86***	0.88***	0.63***	0.81***
Strength of red (*S*_red_)	0.60***	0.89***	0.55***	0.16ns	0.03ns	0.80***	0.33*	0.67***
Strength of blue (*S*_blue_)	0.70***	0.35ns	0.63***	0.43***	0.66***	0.03ns	0.43**	0.16ns

The highest *r* ^2^ for each species‐tissue combination is highlighted in bold.

a indices that were ln transformed.

Absorbance of *S. oleraceus*,* O. italica* and *M. moricandioides* comparisons were also ln transformed. Significance after *Bonferroni's* correction for multiple tests: *p <* *.05; **p <* *.01; ***p <* *.001; ns = nonsignificant.

The relationship between anthocyanin concentration estimated with the biochemical method and *H*,* H*_SC_, and *B* calculated from spectral reflectance data was weak or nonsignificant for most species and tissues, except for *B. officinalis* pedicels and *S. littorea* calyces, with a moderate‐high coefficient of determination (Table 4). Similar results were found with *C* and *C*_SC_ indices, with only a moderate relationship in *S. littorea* petals. In general, indices relating to anthocyanin content (i.e., *AC*_CD_, *AC*_CR_, *AC*_CB_, *R:G*_R_, *R:G*_I_, and *mACI*) showed good performance of the regression model in pedicels and petals of *B. officinalis* (higher *r* ^2^ = .94 and .73, respectively), *S. littorea* petals (*r* ^2^ = .79) and *S. oleraceus* stems (*r* ^2^ = .72) and a moderate performance in *S. littorea* calyces (*r* ^2^ = .61) and *M. sylvestris* petals (*r* ^2^ = .54). *S*_green_ and *S*_red_ showed similar linear relationships to those found in anthocyanin content indices, except for *S*_red_ of *B. officinalis* petals that was not significant. Conversely, *S*_blue_ showed mostly nonsignificant relationships in all species. Based on *r* ^2^, the indices that showed the strongest relationship with anthocyanin concentrations in each species and plant tissues are: *R:G*_I_, *AC*_CR_, *AC*_CD_, *AC*_CB_, *S*_green_, and *mACI* (Table 4).

**Table 4:** Coefficient of determination (*r* ^2^) and statistical significance of linear regressions between relative anthocyanin concentration and spectral reflectance indices

	*B. officinalis*	*B. officinalis*	*M. sylvestris*	*S. littorea*	*S. littorea*	*S. oleraceus* a
Indices	Petals	Pedicels	Petals	Petals	Calyces	Stems
Hue (*H*)	0.01ns	0.66***	0.09ns	0.19ns	0.45***^,^ a	0.32*
Hue‐segment classification (*H*_SC_)	0.18ns	0.86***	0.25ns^,^ a	0.66***	0.52***	0.19ns^,^ a
Brightness (*B*)	0.25ns	0.66***	0.22ns	<0.01ns	0.05ns	0.38**^,^ a
Chroma (*C*)	0.41*	0.37ns	0.15ns	0.60***	<0.01ns^,^ a	0.26*
Chroma‐segment classification (*C*_SC_)	0.09ns	0.37ns	0.32*	0.30*	0.34*	0.03ns
Anthocyanin content‐chroma difference (*AC*_CD_)	0.13ns	0.69***	0.10ns	0.77***	0.61***	0.67***
Anthocyanin content‐chroma ratio (*AC*_CR_)	0.55***	0.72***	0.54***	0.75***	0.45**	0.63***^,^ a
Anthocyanin content‐chroma basic (*AC*_CB_)	0.61***	0.81***^,^ a	0.43**^,^ a	0.79***^,^ a	0.56***^,^ a	0.63***^,^ a
Red:green ratio (*R:G*_R_)	0.69***	0.85***	0.42***^,^ a	0.75***^,^ a	0.60***	0.57***
Red:green index (*R:G*_I_)	0.71***	0.94***	0.43**^,^ a	0.76***^,^ a	0.53***	0.70***^,^ a
Modified anthocyanin content index (*mACI*)	0.73***	0.87***^,^ a	0.43**^,^ a	0.75***^,^ a	0.28*	0.60***^,^ a
Strength of green (*S*_green_)	0.54***	0.78***	0.50***	0.77***	0.57***	0.72***
Strength of red (*S*_red_)	0.16ns	0.84***	0.41**	0.70***	0.58***	0.69***
Strength of blue (*S*_blue_)	0.51**	0.39ns	<0.01ns	0.01ns	0.34*	0.01ns

The highest *r* ^2^ for each species‐tissue combination is highlighted in bold.

a Indices that were ln transformed.

Absorbance of all *S. oleraceus* comparisons was also ln transformed. Significance after *Bonferroni's* correction for multiple tests: *p <* *.05; **p <* *.01; ***p <* *.001; ns = nonsignificant.

**Figure 3:**
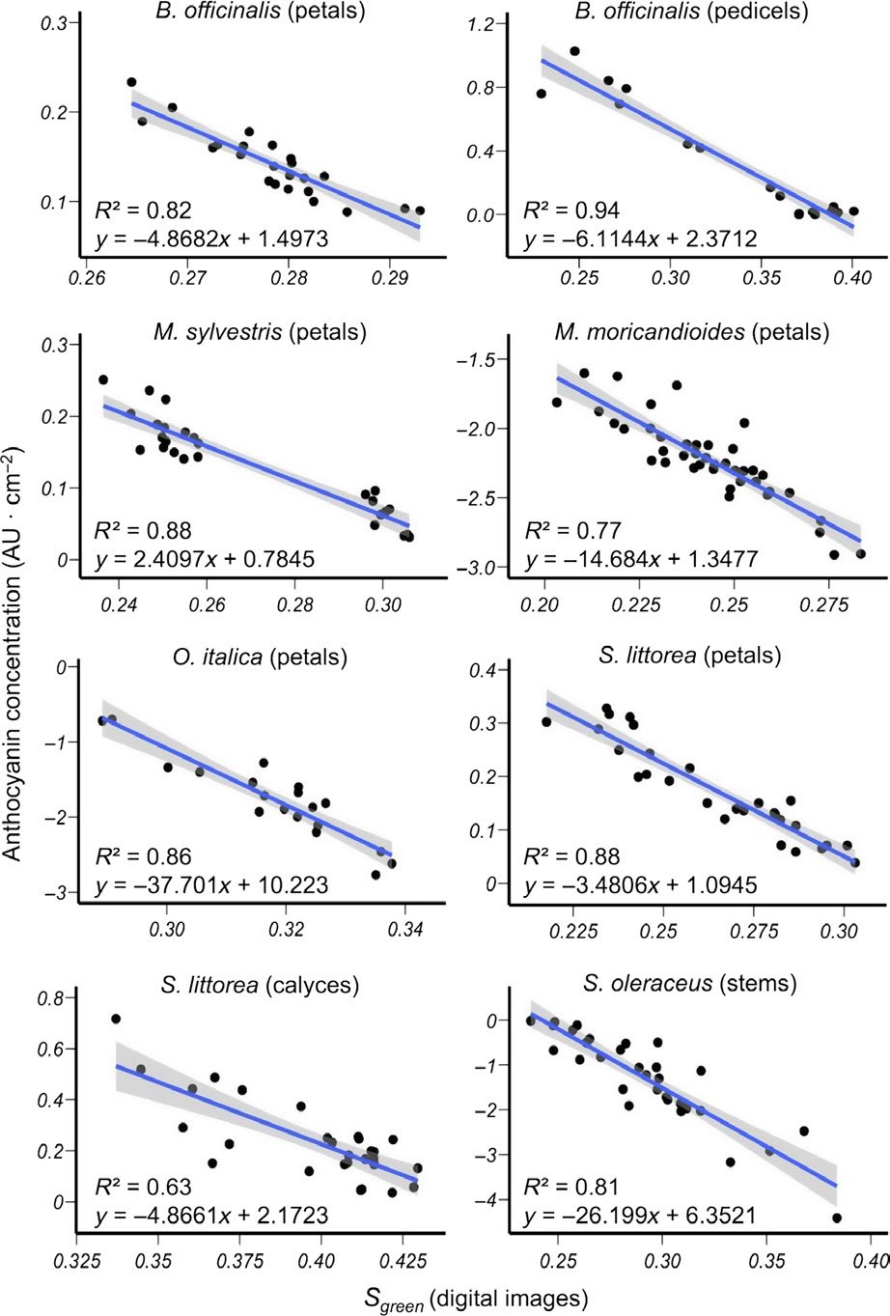
Relationship between relative anthocyanin concentration estimated from the biochemical method and *S*_green_ calculated from digital images in various species and tissues. Statistics of the regression models and the best‐fit linear regression lines with 95% confidence intervals (shaded) are shown. Absorbance values of *M. moricandioides*,* O. italica* and *S. oleraceus*, were log transformed (see Table 3)

**Figure 4:**
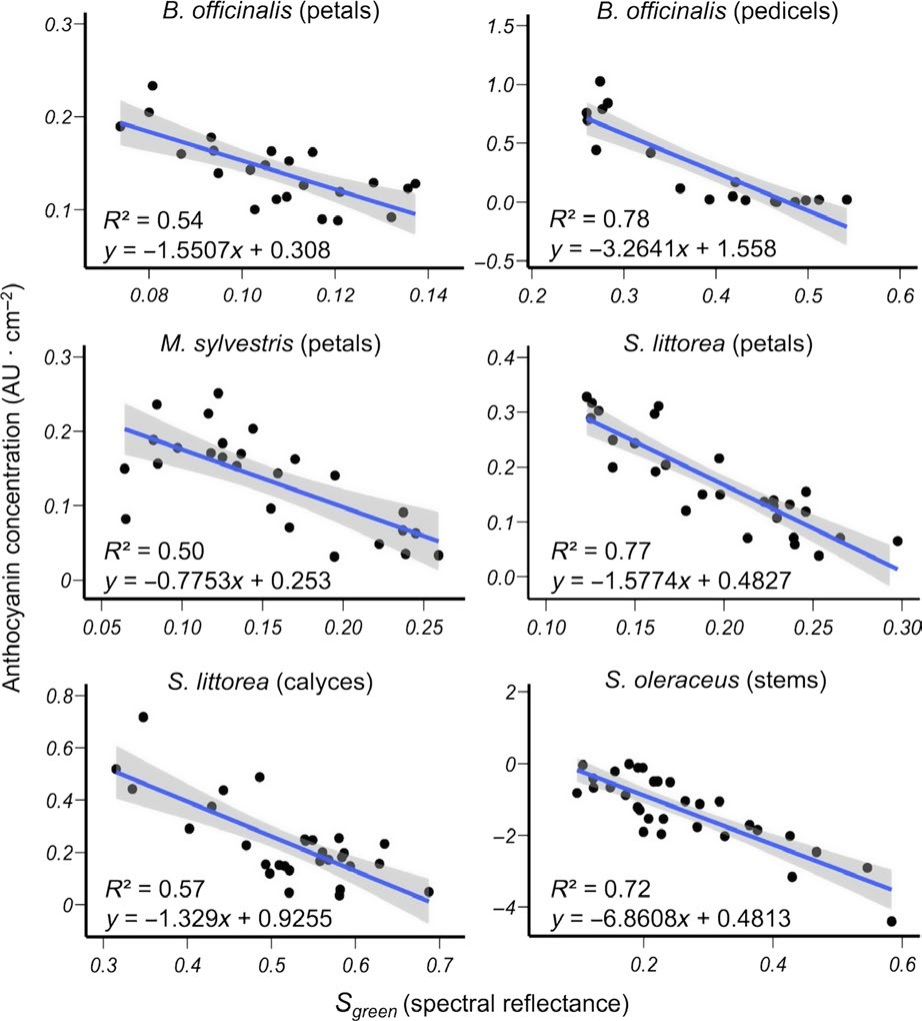
Relationship between relative anthocyanin concentration estimated from biochemical method and *S*_green_ calculated from spectral reflectance in various species and tissues. Statistics of the regression models and the best‐fit linear regression lines with 95% confidence intervals (shaded) are shown. Absorbance values of *S. oleraceus* were log transformed (see Table 4)

In general, digital image method showed slight better accuracy than spectral reflectance method in estimating anthocyanin concentrations (Table S2). Mean NRMSE was higher in all plant samples, ranging from 8.6% to 13.1% in digital image estimations (*B. officinalis* pedicels and *S. oleraceus* stems, respectively), and from 13.5% to 22.5% in spectral reflectance estimations (*S. littorea* petals and *M. sylvestris* petals, respectively). This high accuracy of the model's predictions can be observed in Figures 3 and 4, which show the relationship between anthocyanin concentration from biochemical method and *S*_green_ from digital image and spectral reflectance data. An exception was found in *S. littorea* calyces, which showed nearly similar NRMSE in both methods (Table S2). Finally, the validation of regression model of anthocyanin amount in petals of *S. littorea* using independent samples showed a high relationship between observed and predicted values (*r* ^2^ = .90, *p *<* *.0001), with the slope of the regression model that was not significantly different from a slope of 1 (slope = 0.923, *t *=* *−0.225, *df* = 26, *p *=* *.25; Figure S2).

## 4 DISCUSSION

We propose several digital image‐based color indices that accurately predict anthocyanin concentration in species with a diversity of anthocyanins or anthocyanins plus chlorophyll. Indices related to hue and brightness showed variable results depending on species and the plant tissue analyzed, whereas chroma and anthocyanin content indices yielded a reasonable goodness‐of‐fit score in most samples. This result is not surprising given that within species variation in pigment concentration usually affects indices based on variation between different areas of color spectra or RGB channels (Curran, 1989; Gitelson, Keydan, & Merzlyak, 2006; Gonnet, 1999). Specifically, indices that simply stated the ratio of the G channel over the R and/or B channels (i.e., *AC*_CR_, *AC*_CB_ and *R:G*_R_) yielded the best results in terms of coefficient of determination. Similarly, among indices related to strength of each RGB channel, the best result was found with *S*_green_. As anthocyanin pigments show absorption in the green region of the spectrum, and reflect red, blue, and purple wavelengths (Gitelson, Merzlyak, & Chivkunova, 2001; van der Kooi et al., 2016); it follows that variation in the G channel is particularly effective for estimating anthocyanin concentration using digital images. In this way, Mizunuma et al. (2014) showed the suitability of digital images to discriminate leaf colors and estimate chlorophyll concentration. Chlorophylls absorb in the red region of spectra, and accordingly, indices accounting for the R channel showed the best performance (Mizunuma et al., 2014).

Methods based on spectral reflectance can reliably estimate anthocyanin pigments in leaves, fruits, and stems (Gamon & Surfus, 1999; Gitelson et al., 2009; Gould, Dudle, & Neufeld, 2010), yet often require destructive sampling. Here, we show that this type of data is also suitable to estimate anthocyanin concentration in petals and other photosynthetic tissues such as pedicels. In fact, most anthocyanin content indices and *S*_green_ calculated from reflectance data had moderate to good ability in predicting the anthocyanin concentration. Nevertheless, our major finding is that digital images can produce reliable estimates of quantitative variation in anthocyanin concentration in photosynthetic and nonphotosynthetic tissues. In fact, both spectral reflectance and digital image methods are based on reflectance of light reaching the plant tissue. The spectrophotometer may provide increased spectral resolution than the digital camera, especially when the characteristics of the camera or lens are low quality (Garcia, Dyer, Greentree, Spring, & Wilksch, 2013; Kendal et al., 2013; Mizunuma et al., 2014). However, the recent advance of camera optics and sensors and the increasing freely available open‐source softwares for image processing allow efficient acquisition of data appropriate for a precise color determination (Akkaynak et al., 2014; Garcia et al., 2014; Troscianko & Stevens, 2015) affording accurate quantification of pigment without destructive sampling of the plant tissue.

In most species and tissues, estimating anthocyanin concentration by digital images showed similar or slightly better performance than using spectral reflectance. In petals of *M. sylvestris*, this difference was more pronounced, which might be due to their striped pigmentation pattern. Similarly, previous studies reported high effectiveness of color captures using digital images when measures are carried out in biological material with nonuniform colors or texture (Gómez & Liñán‐Cembrano, 2016; Kendal et al., 2013; Pike, 2011; Stevens et al., 2007). In this vein, Garcia et al. (2014) compared digital images with spectrophotometer data in analyzing petal color of eight species with variable pigmentation patterns and found that when the pattern is complex, the spectrophotometer would potentially underestimate spectral signal variability. In our study, the spectrometer probe holder has a relatively small sampling area, which could lead to a different spectral measurement depending if, by chance, it was positioned on a light or dark stripe or patch. This explains why samples with similar anthocyanin concentration showed very different values of *S*_green_ calculated form spectral reflectance (Figure 4). This problem can be solved by measuring reflectance in multiple points of the sample (e.g., Garcia et al., 2014), but this clearly increases the time spent analyzing one sample, particularly when it is compared with the time needed to take a single photograph. Although we do not have reflectance data of petals of *O. italica*, which show a spotted pattern, the digital image method showed a high correlation with anthocyanin concentration. Conversely, in striped calyces of *S. littorea*, the digital image method fails to increase both *r* ^2^ and NMRSE compared to the spectral reflectance method. In petals of *O. italica* and *M. sylvestris*, anthocyanins accumulate in epidermal cells, whereas in *S. littorea* calyces, the anthocyanins are also stored in basal cells of the trichomes (Del Valle et al., 2015). The cylindrical structure of these cells could cause a discrepancy between the amount of anthocyanin estimated by digital images (in two dimensions) and the concentration analyzed with the biochemical method.

Although our digital image methodology has numerous advantages, there are some limitations. For example, when anthocyanins are not homogeneously distributed throughout a three‐dimensional structure, digital images may not accurately predict the anthocyanin concentration because single digital images can only capture two‐dimensional plane of data. Digital photography may also fail in cases when the cells of the measured surface are irregular. Cell shape has been shown to change the perceived color (Glover, 2007), which would cause errors when estimating anthocyanin concentration from digital images. In addition, other characteristics such as the presence of waxes, cell wall thickness, or pigment location may also affect the observed color (Kay, Daoud, & Stirton, 1981; van der Kooi et al., 2016). Finally, our methodology performs well when analyzing variation among samples in concentration of the same anthocyanin type (e.g., cyanidin derivatives). However, when samples differ in the type of pigment (e.g., anthocyanin vs. carotenoid), type of anthocyanin (cyanidin vs. pelargonidin), or in their biochemical modifications (glycosylation & acylation; Merken & Beecher, 2000; Nogales‐Bueno, Baca‐Bocanegra, Rodríguez‐Pulido, Heredia, & Hernández‐Hierro, 2015), digital images will capture the visible color, but there will be no associated changes in the raw anthocyanin concentration. In these cases, other indices may show improved performance, but require further study.

In conclusion, we have demonstrated that digital images bring new opportunities to accurately quantify anthocyanin concentration in both floral and vegetative plant tissues. The principal advantages are efficiency, totally noninvasive, applicable to patterned tissues, and useful for plant samples of any size and shape. We recommend to use *AC*_CR_, *AC*_CB_ or *S*_green_ indices because of their simplicity and performance in most species and tissues, including samples with red, pink, and blue colors. In addition, the selection of the most appropriate index with complex tissues or sample colors should be tested in a subset of samples following the pipeline described here. Our method could be particularly useful for studies attempting to unravel the ecological interactions and evolutionary forces molding flower color variation. Variation in floral anthocyanin content or pattering may be under selection by pollinators (Ortiz‐Barrientos, 2013; Sletvold, Trunschke, Smit, Verbeek, & Ågren, 2016) or nonpollinators alike (Narbona, Wang, Ortiz, Arista, & Imbert, 2017; Strauss & Cacho, 2013; Strauss & Whittall, 2006). Similarly, the accumulation of anthocyanins in vegetative organs or tissues such as leaves, stems, or pedicels is also influenced by direct or indirect selection of biotic and abiotic factors (Cooney, Schaefer, Logan, Cox, & Gould, 2015; Gould et al., 2010; Menzies et al., 2016). In order to estimate the fitness consequences of such anthocyanin variation (e.g., Del Valle et al., 2015; Sletvold et al., 2016), one must employ an efficient, noninvasive method such as digital photography.

## DATA ACCESSIBILITY

Data for the studied species have been deposited in the Dryad repository: https://doi.org/10.5061/dryad.kb552. Two samples, representing the lightest and darkest spectral reflectance of each species, were deposited in the Floral Reflectance Database (http://www.reflectance.co.uk/).

## CONFLICT OF INTEREST

None declared.

## AUTHORS’ CONTRIBUTIONS

EN conceived the ideas and designed the study; JCD, EN, and AG collected data. JCD, EN, and JBW conducted analysis of data and developed new indices for anthocyanin estimation; EN, JCD, JBW, and MB wrote the article. All authors contributed critically to the drafts and gave final approval for publication.
