# Supplementary figures and images for: Digital photography provides a fast, reliable, and noninvasive method to estimate anthocyanin pigment concentration in reproductive and vegetative plant tissues
Source: Ecol Evol. 2018 Feb 16;8(6):3064–76. doi: 10.1002/ece3.3804 (PMC5869271; doi:10.1002/ece3.3804)

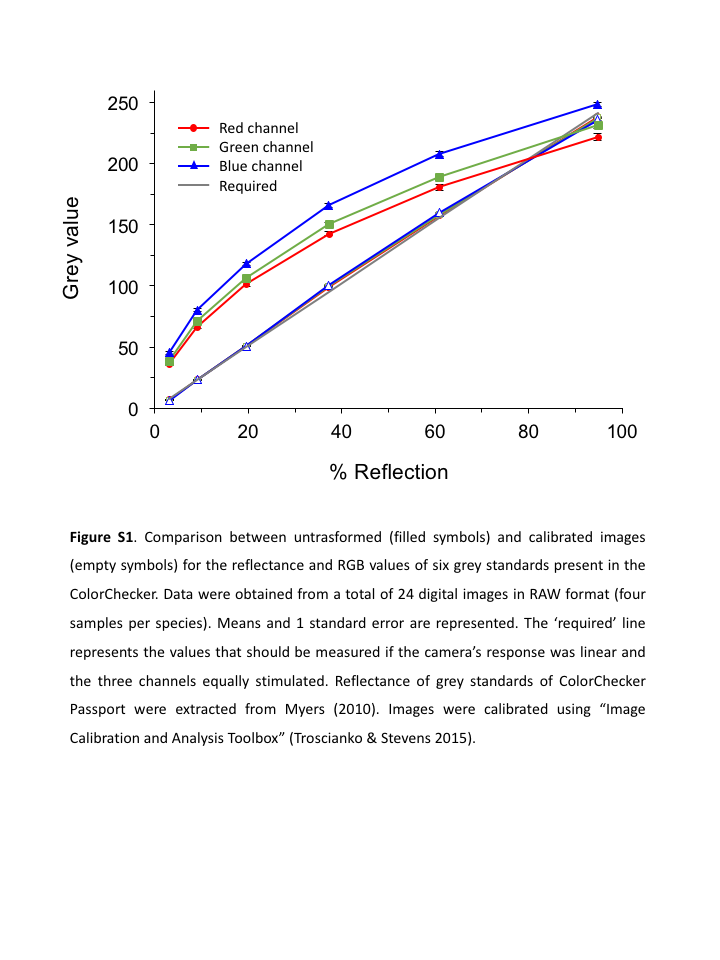

Supplement: Supplementary file 1 [file ECE3-8-3064-s001.tiff]

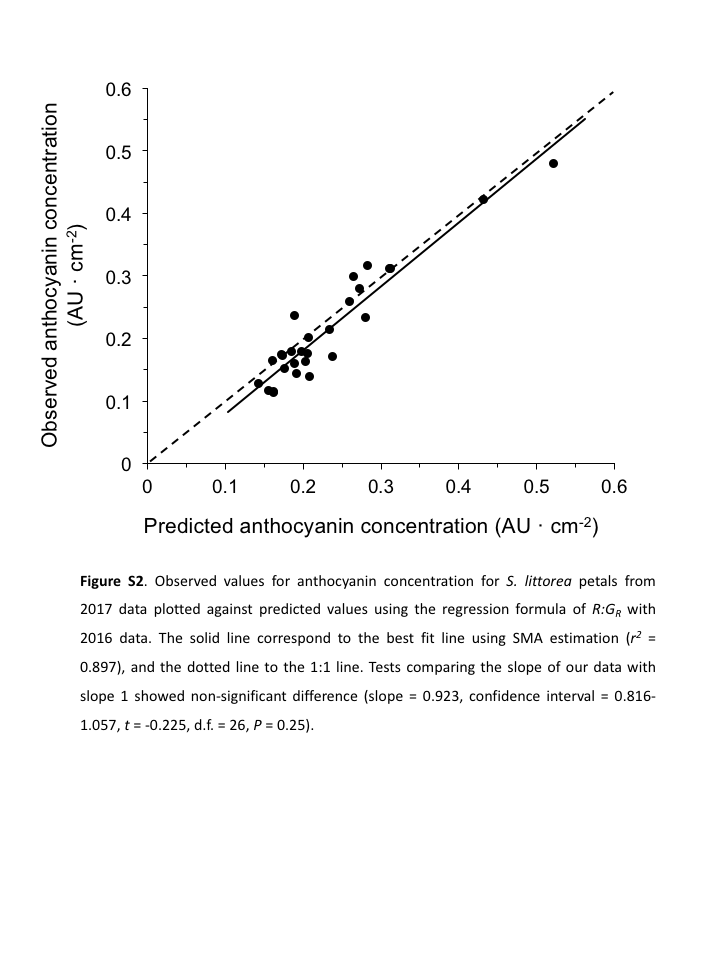

Supplement: Supplementary file 2 [file ECE3-8-3064-s002.tiff]
