# Supplementary material for: Digital photography provides a fast, reliable, and noninvasive method to estimate anthocyanin pigment concentration in reproductive and vegetative plant tissues
Source: Ecol Evol. 2018 Feb 16;8(6):3064–76. doi: 10.1002/ece3.3804 (PMC5869271; doi:10.1002/ece3.3804)
Supplement: Supplementary file 3 [file ECE3-8-3064-s003.docx]

| **Table S1.** ANOVAs comparing values of red, green and blue channels obtained after image calibration using two *versus* six grey standards of the ColorChecker Passport. | | | | | | | | |
| --- | --- | --- | --- | --- | --- | --- | --- | --- |
|  | Red channel | |  | Green channel | |  | Blue channel | |
|  | *F* | *P* |  | *F* | *P* |  | *F* | *P* |
| *Sonchus littorea* | 0.280 | 0.601 |  | 0.116 | 0.736 |  | 0.223 | 0.640 |
| *Silene oleraceus* | 0.017 | 0.898 |  | 0.020 | 0.888 |  | 0.048 | 0.828 |
| We randomly chose 15 samples of each *S. littorea* (petals) and *S. oleraceus* (stems). Images were calibrated using “Image Calibration and Analysis Toolbox” (Troscianko & Stevens 2015). | | | | | | | | |

**Table S2**. Normalized root-mean-square errors (NRMSE) for anthocyanin content-chroma basic (*AC_CB_*), anthocyanin content-chroma ratio (*AC_CR_*), red:green ratio (*R:G_R_*) and strength of green (*S_green_*) indices using spectral reflectance and digital images methods to estimate anthocyanin concentrations. The mean among the four indices is also shown.

|  |  |  |  |  | | |  | | |  | NRMSE (%) | | | | |  | |
| --- | --- | --- | --- | --- | --- | --- | --- | --- | --- | --- | --- | --- | --- | --- | --- | --- | --- |
|  |  | *AC_CB_* | | | *AC_CR_* | | | | *R:G_R_* | | | | *S_green_* | | Mean | | |
| Species | Plant part | Spectra | Image | | Spectra | | | Image | | Spectra | | Image | Spectra | Image | Spectra | | Image |
| *Borago officinalis* | Petals | 17.6 | 9.9 | | | 16.4 | | 10.2 | | 14.0 | | 20.1 | 18.5 | 10.1 | 16.6 | 12.6 | |
|  | Pedicels | 14.1 | 8.7 | | 17.6 | | | 8.5 | | 13.8 | | 9.1 | 15.7 | 8.0 | 15.3 | 8.6 | |
| *Malva sylvestris* | Petals | 24.4 | 11.9 | | 20.5 | | | 12.7 | | 23.6 | | 11.7 | 21.3 | 12.5 | 22.5 | 12.2 | |
| *Silene littorea* | Petals | 13.6 | 9.4 | | 13.1 | | | 9.7 | | 13.8 | | 10.3 | 13.5 | 9.6 | 13.5 | 9.8 | |
|  | Calyces | 15.2 | 16.0 | | 16.4 | | | 17.0 | | 14.9 | | 16.4 | 15.6 | 16.6 | 15.6 | 16.6 | |
| *Sonchus oleraceus* | Stems | 15.2 | 12.4 | | 17.1 | | | 14.1 | | 18.9 | | 12.2 | 18.6 | 13.6 | 17.5 | 13.1 | |
