# Supplementary material for: Digital photography provides a fast, reliable, and noninvasive method to estimate anthocyanin pigment concentration in reproductive and vegetative plant tissues
Source: Ecol Evol. 2018 Feb 16;8(6):3064–76. doi: 10.1002/ece3.3804 (PMC5869271; doi:10.1002/ece3.3804)
Supplement: Supplementary file 4 [file ECE3-8-3064-s004.docx]

**Supplemental Methods**

**Data S1:** Model validation using independent data from *S. littorea* petals.

In spring 2017, one year after the initial measurements of *S. littorea*, biochemical and digital image methods were applied in another 28 plants growing in the greenhouse, using the same pre- and post-image processing that previous data. We calculated the predicted amount of anthocyanins using the regression model of the dataset from 2016 with the best digital image indices (*R:G_R_*, see results). These predicted values were regressed with the measured amount of anthocyanins in the new dataset (Piñeiro *et al.* 2008), and the slope if the regression line against the 1:1 line were compared using Standardised Major Axis estimation (SMA; Warton *et al*. 2006). SMA analysis was conducted in the R-package smatr3 (Warton *et al*. 2012).

**References**:

Piñeiro, G., Perelman, S., Guerschman, J.P. & Paruelo, J.M. (2008). How to evaluate models: Observed vs. predicted or predicted vs. observed? *Ecological Modelling*, **216**, 316–322.

Warton, D.I., Wright, I., Falster, D. & Westoby, M. (2006). Bivariate line-fitting methods for allometry. *Biological Review*, **81**, 259–291.

Warton, D.I., Duursma, R.A., Falster, D.S. & Taskinen, S. (2012). smatr 3–an R package for estimation and inference about allometric lines. *Methods in Ecology and Evolution*, **3**, 257–259.
